# Construction of an *Escherichia coli* chassis for efficient biosynthesis of human-like *N*-linked glycoproteins

**DOI:** 10.3389/fbioe.2024.1370685

**Published:** 2024-03-20

**Authors:** Zixin Bao, Yuting Gao, Yitong Song, Ning Ding, Wei Li, Qiong Wu, Xiaomei Zhang, Yang Zheng, Junming Li, Xuejun Hu

**Affiliations:** ^1^ Academic Centre for Medical Research, Medical College, Dalian University, Dalian, China; ^2^ Dalian Key Laboratory of Oligosaccharide Recombination and Recombinant Protein Modification, Dalian, China; ^3^ Department of Clinical Laboratory, Yantai Yuhuangding Hospital, Yantai, China

**Keywords:** *Escherichia coli* chassis, *N*-glycosylation, glycoprotein, sialylation, sialic acid

## Abstract

The production of *N*-linked glycoproteins in genetically engineered *Escherichia coli* holds significant potential for reducing costs, streamlining bioprocesses, and enhancing customization. However, the construction of a stable and low-cost microbial cell factory for the efficient production of humanized *N*-glycosylated recombinant proteins remains a formidable challenge. In this study, we developed a glyco-engineered *E. coli* chassis to produce *N*-glycosylated proteins with the human-like glycan Gal-β-1,4-GlcNAc-β-1,3-Gal-β-1,3-GlcNAc-, containing the human glycoform Gal-β-1,4-GlcNAc-β-1,3-. Our initial efforts were to replace various loci in the genome of the *E. coli* XL1-Blue strain with oligosaccharyltransferase PglB and the glycosyltransferases LsgCDEF to construct the *E. coli* chassis. In addition, we systematically optimized the promoter regions in the genome to regulate transcription levels. Subsequently, utilizing a plasmid carrying the target protein, we have successfully obtained *N*-glycosylated proteins with 100% tetrasaccharide modification at a yield of approximately 320 mg/L. Furthermore, we constructed the metabolic pathway for sialylation using a plasmid containing a dual-expression cassette of the target protein and CMP-sialic acid synthesis in the tetrasaccharide chassis cell, resulting in a 40% efficiency of terminal α-2,3- sialylation and a production of 65 mg/L of homogeneously sialylated glycoproteins in flasks. Our findings pave the way for further exploration of producing different linkages (α-2,3/α-2,6/α-2,8) of sialylated human-like *N*-glycoproteins in the periplasm of the plug-and-play *E. coli* chassis, laying a strong foundation for industrial-scale production.

## Introduction

In 2002, Wacker et al. achieved a significant breakthrough in glycoprotein production in *Escherichia coli* by introducing the receptor protein and *pgl* gene cluster from *Campylobacter jejuni* to carry out protein glycosylation (Wacker et al., 2002). Over the subsequent 2 decades, researchers have employed various glycosyltransferases and oligosaccharyltransferases (OSTase) to produce humanized *N*-glycosylated recombinant proteins, as well as to facilitate pathogenic bacterial oligosaccharide vaccine production in *E. coli* (Hug et al., 2011; Valderrama-Rincon et al., 2012; Harding et al., 2019; Williams et al., 2023). Currently, there are two predominant pathways utilized for *N*-glycosylation of proteins in *E. coli*: the non-OSTase-dependent system that utilizes *N*-glycosyltransferase (NGT) in the cytoplasm, and the *C. jejuni* PglB-based OST dependent system in the periplasm. In 2019, Tytgat et al. established a human-like *N*-glycan trisaccharide Neu5Ac-α-2,3-Gal-β-1,4-Glc- synthetic pathway using the *N*-glycosyltransferase from *Actinobacillus pleuropneumoniae* (ApNGT), achieving a glycosylation efficiency of 20%–62% (Tytgat et al., 2019). This glycan is shorter than typical human *N*-glycosylated antennae glycans, which alleviates certain challenges such as steric hindrance during recombinant protein folding, and can be further extended into diverse human-like sialylated glycans (Mitra et al., 2006; Shental-Bechor and Levy, 2009; Mansell et al., 2013; Abukar et al., 2024). However, this extension is limited by the low assembly efficiency of oligosaccharides, suboptimal specific activity against heterologous substrates, and the requirement for the costly and unstable donor Neu5Ac (Prabhu et al., 2021). In 2012, DeLisa’s group utilized the O antigen synthesis mechanism, the WecA enzyme, in combination with Alg14/13/12 enzymes from *Saccharomyces cerevisiae*, to develop a “one-step” method to obtain *N*-glycoproteins with a Man3G1cNAc2 glycan in *E. coli* (Valderrama-Rincon et al., 2012). By 2018, they optimized yeast-derived glycan synthesis-related enzymes, increasing the yield of human-like pentasaccharides by nearly 50-fold and the glycosylated receptor proteins by over 10-fold, to 14 mg/L (Glasscock et al., 2018). During this period, Phillips and Hug and their respective co-workers synthesized a human-like tetrasaccharide Gal-β-1,4-GlcNAc-β-1,3-Gal-β-1,3-GlcNAc- (Gal-β-1,4-GlcNAc-β-1,3R being one of the terminal composition modes of human glycoepitopes) in the periplasm of *E. coli*. This was then linked to receptor proteins with 40% glycosylation efficiency (Phillips et al., 2000; Hug et al., 2011). Ding et al. established an equally efficient human-like *N*-glycosylated protein synthesis system in the *E. coli* periplasm using an auto-induction method, achieving up to 100% glycosylation efficiency (Ding et al., 2017). Recently, a dual-plasmid-based sialic acid synthesis and activation pathway was constructed in *E. coli* to produce human-like glycans terminally modified by a sialic acid residue; however, there is an urgent need to improve the expression level of *N*-glycoproteins (Zhu et al., 2020). This is attributed to the inherent instability of multi-plasmid systems (Silva et al., 2012; San Millan et al., 2017), necessitating further exploration.

Efforts to optimize production stability in *E. coli* have encompassed heterologous expression of glycosylation enzymes, metabolic engineering, and attenuation of gene expression (Hug et al., 2011; Ollis et al., 2015; Pratama et al., 2021; Kay et al., 2022; Liu et al., 2023). Integration of the exogenous *N*-glycosylation pathway into the host genome has been a prominent focus to enhance the stability of glycosylation systems in *E. coli*. In 2017, Wright and co-workers introduced the *pgl* gene cluster from *C. jejuni* into the *E. coli* genome, resulting in a 1.8-fold enhancement in *N*-glycoprotein yield, but with an efficiency of glycosylation of only 40% (Strutton et al., 2018). Subsequent integration of the *pgl9* gene cluster (to synthesize GalNAc5(Glc)GlcNAc) and *pglB* into the engineered *N*-glycosylation pathway by the DeLisa group. Targeting two specific sites, O-polysaccharide antigen (O-PS) and enterobacterial common antigen (ECA), resulted in a remarkable 82% glycosylation efficiency, surpassing that of triple-plasmid systems (Yates et al., 2019). However, there has been no systematic evaluation of glycoprotein yield thus far. Optimization of integration of exogenous *N*-glycosylation pathway genes into different genome sites is expected to effectively address the challenge of simultaneously improving glycoprotein yield and modification efficiency.

Studies have demonstrated that protein yields can be enhanced by modifying or introducing supplementary promoter elements, or by adjusting gene copy numbers in the *E. coli* genome (Yates et al., 2019; Jiang et al., 2021; Passmore et al., 2023). Although low gene copy numbers increase the stability of the expression system when acting as substrates or intermediates in the metabolic pathways involved in the normal metabolism of the host, it typically also results in a decrease in the yield of the target protein. In 2021, Jiang’s team constructed a chassis strain optimized for glycan synthesis, utilizing a moderately strong promoter, P11-BCD15, to produce O-PS conjugates (Jiang et al., 2021). This approach significantly enhanced glycosylation efficiency up to 90.7% and increased product yield to 38.6 mg/L. Further research confirmed that integrating the *pgl* gene cluster into the *E. coli* SDB1 genome at 30°C could increase glycosylation efficiency and yield, provided there was more than one copy of PglB available (Terra et al., 2022). A single antenna glycan in humans is known to have at least a pentasaccharide composition and to be naturally sialylated with different modes of α-2,3, α-2,6, or α-2,8 linkages. In this regard, constructing an *E. coli* chassis to explore efficient *N*-glycosylation with α-2,3 linked sialylation holds significant potential in paving the way for more effective and versatile modification of recombinant glycoproteins, thereby enhancing their functionality and applicability.

To establish a stable glyco-engineered *E. coli* chassis, this study involved the tandem integration of glycosyltransferase *lsgCDEF* gene cluster and oligosaccharyltransferase *pglB* in *E. coli* XL1-Blue, replacing the native *ECA* and *nanKETA* gene clusters. The integrated genes were regulated by a range of promoters of differing strengths, then a plasmid encoding target receptor proteins was utilized to produce *N*-glycoproteins modified with the human-like glycan Gal-β-1,4-GlcNAc-β-1,3-Gal-β-1,3-GlcNAc- in the engineered strain. Furthermore, to maximize the production of sialylated *N*-glycoproteins, the *nanKETA* gene cluster was selectively replaced with α-2,3-sialyltransferase and *neuBCA* synthetase genes to compare with the dual-expression cassette plasmid containing the sialylation pathway. This work establishes a plug-and-play *E. coli* chassis for industrial production of homogeneous *N*-glycoproteins modified with specific sialylation linkages to mimic human glycoepitopes.

## Materials and methods

### Construction of plasmids and biological pathways

All strains used in this study are listed in Table 1; plasmids and primers are summarized in Supplementary Table S1. Molecular cloning and plasmid construction were carried out in *E. coli* Top10. The vectors pIG6-FN3-Gly-1, pIG6-Sia, and pC15-plsg were used for cloning and subcloning. The FN3 sequence in pIG6-FN3-Gly-1 was replaced with the optimized Fn3 variant of a mesothelin-targeting antibody, Fn3 3.4.4 (Sirois et al., 2020), resulting in the plasmid pIG6-FM-Gly. The gene sequence encoding 2,3SiaTpph (A151D) was inserted into the vector pIG6-Sia, yielding pIG6-pst3. Modification of the pC15-plsg plasmid involved the removal of the *wecA* gene and the addition of a kanamycin resistance (Kan^R^) cassette flanked by FRT sites from the pKD13 plasmid upstream of the arabinose promoter, yielding pC15-plsgΔw.

**TABLE 1:** Strains used in this study.

Strains	Characteristics	Source
XL1-Blue	endA1 gyrA96 (nalR) thi-1 recA1 relA1 lac glnV44 F‘ [Tn10 proAB+ lacIq Δ(lacZ)M15] hsdR17 (rK- mK+)	Agilent Technologies, Inc.
XΔK	*E. coli* XL1-BlueΔnanKETA	This study
XEA	XL1-Blue derivate, ΔECA:Ara-pglB-pglK-lsgCDEF	This study
XKA	XL1-Blue derivate, ΔnanKETA:Ara-pglB-pglK-lsgCDEF	This study
XL-G	XL1-Blue harboring plasmids pIG6-FM-Gly and pC15-plsgΔW	This study
XEA-G	XEA harboring plasmids pIG6-FM-Gly	This study
XKA-G	XKA harboring plasmids pIG6-FM-Gly	This study
XE1	XL1-Blue derivate, ΔECA:Ptac-pglB-pglK-lsgCDEF	This study
XE2	XL1-Blue derivate, ΔECA:PlacUV5-Ptac-pglB-pglK-lsgCDEF	This study
XE3	XL1-Blue derivate, ΔECA:Ptac-PlacUV5-Ptac-pglB-pglK-lsgCDEF	This study
XE1-G	XE1 harboring plasmids pIG6-FM-Gly	This study
XE2-G	XE2 harboring plasmids pIG6-FM-Gly	This study
XE3-G	XE3 harboring plasmids pIG6-FM-Gly	This study
XK1	XL1-Blue derivate, ΔnanKETA:Ptac-pglB-pglK-lsgCDEF	This study
XK2	XL1-Blue derivate, ΔnanKETA:PlacUV5-Ptac-pglB-pglK-lsgCDEF	This study
XK3	XL1-Blue derivate, ΔnanKETA:Ptac-PlacUV5-Ptac-pglB-pglK-lsgCDEF	This study
XK1-G	XK1 harboring plasmids pIG6-FM-Gly	This study
XK2-G	XK2 harboring plasmids pIG6-FM-Gly	This study
XK3-G	XK3 harboring plasmids pIG6-FM-Gly	This study
XE2ΔK	XE2 derivate, ΔnanKETA	This study
XE2ΔK-pst3	XE2 derivate, ΔnanKETA:P-pst3	This study

### Chromosomal integration of DNA

Linear DNA was produced for integration using plasmid DNA as a PCR template. The DNA was then electrotransformed into the target bacterial strain expressing the λ-Red recombinase using pKD46. The bacteria were incubated overnight at 30°C on LB agar plates containing 100 μg/mL ampicillin (Amp) and 50 μg/mL Kan for selection. Colonies were transferred to antibiotic-free medium and grown at 42°C for 12 h to remove the pKD46 plasmid. The bacteria were streaked onto LB agar plates containing 100 μg/mL Amp or 50 μg/mL Kan. The Amp^R^/Kan^R^ colonies were collected and the successful integration of target genes was confirmed using colony PCR and nucleotide sequencing (Genewiz, Shanghai, China).

### Protein expression and purification

Colonies were inoculated into 3 mL of LB broth (with appropriate antibiotics) for overnight cultivation at 37°C and 220 rpm. The next day, 100 μL of the culture was transferred into 500 mL of medium in a shake flask and cultured at 37°C until the cell density reached an optical density at 600 nm (OD_600_) of approximately 0.6. Isopropyl β-D-1-thiogalactopyranoside (IPTG; 0.5 mM) and/or arabinose (0.2% (w/v)) were added and the temperature was reduced to 25°C. Cell densities (OD_600_) of each culture were regularly measured over 48 h, and three biological experiments were performed. After culturing, the cells were harvested and target proteins were purified as previously described (Ding et al., 2017).

### Immunoblot analysis

The purified FM proteins (sialylated, non-sialylated, and non-glycosylated) were separated on a 15% SDS-PAGE gel and subjected to Coomassie brilliant blue staining, and immunodetection with monoclonal anti-flag M1 antibody (Sigma-Aldrich) and the Horseradish peroxidase (HRP) -labeled rabbit anti-mouse IgG (Invitrogen). Proteins with different glycosylation states were also detected with ECA-HRP and MAL-II-HRP lectins. The blots were visualized using the ChemidocTM XRS+ system (Bio-Rad) and analyzed with the Image Lab software. These data were derived from at least three replicate experiments.

### MALDI-TOF detection

To conduct detection, 2 μL of sample was mixed with 2 μL of 2% trifluoroacetic acid (TFA) and 2 μL of matrix solution, which comprises 2,5-DHAP. Then, 1 μL of the mixture was spotted onto the MALDI metal target and air-dried at room temperature. Detection was carried out by Beijing Omicss Biotechnology Co., Ltd using a Bruker ultrafleXtremeTM MALDI-TOF/TOF mass spectrometer. The experiment was repeated three times.

## Results

### Design and construction of glyco-engineered *E. coli* chassis for biosynthesis of human-like tetrasaccharide glycan

**FIGURE 1:**
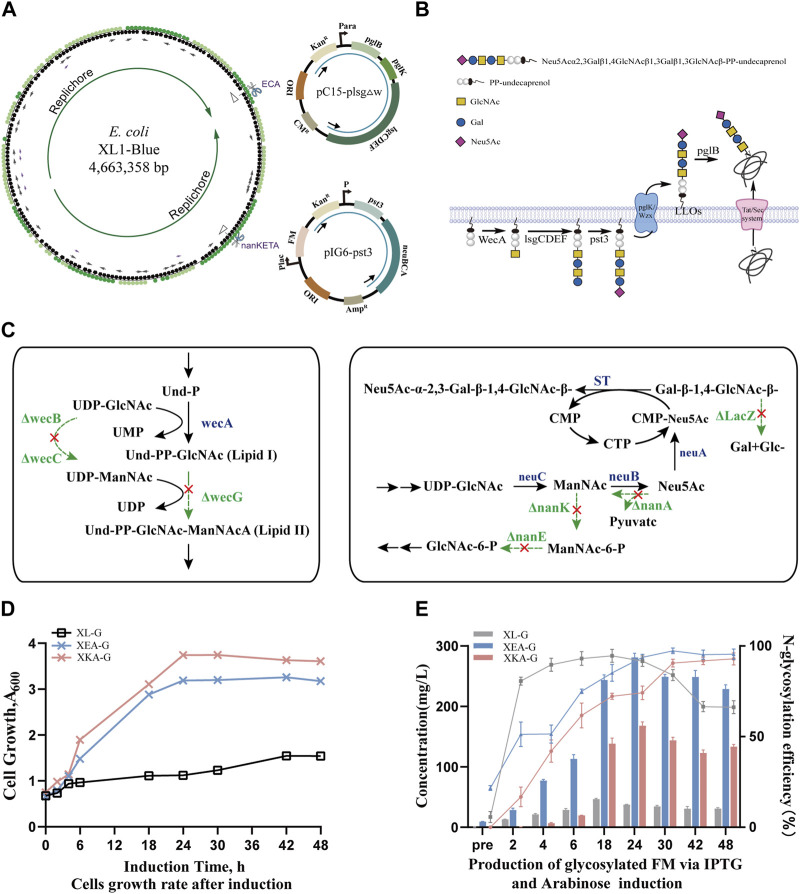
Construction and production of human-like glyco-engineered *E. coli* strains. **(A)** A circular diagram of the *E. coli* XL1-Blue strain chromosome depicts the genomic integrations at ECA and *nanKETA* sites, alongside their corresponding replacement fragments in plasmids pC15-plsgΔw and pIG6-pst3. Plasmid pC15-plsgΔw contains the genes of oligosaccharide transferase and the required enzymes for tetrasaccharide biosynthesis; dual-expression cassette plasmid pIG6-pst3 contains genes encoding sialyltransferase and the enzymes of CMP-sialic acid biosynthesis. **(B)** A schematic of the biosynthesis pathway for *N*-glycoprotein with terminal sialylation *in vivo*. The O-antigen synthetase (WecA) from *E. coli*, glycosyltransferases (LsgCDEF) from *H. influenzae,* and PglK and oligosaccharide transferase (PglB) from *C. jejuni* are combined with sialyltransferase (Pst3) to transfer oligosaccharides to target proteins in the periplasm. **(C)** Metabolic pathways of knockout sites in the genome. On the left is the competing pathway of the ECA locus, on the right is the CMP-sialic acid synthesis pathway, and the bypass pathway of the *nanKETA* locus. **(D)** Cell growth curves of the glyco-engineered strain expressing glycosylated recombinant proteins modified with human-like Gal-β-1,4-GlcNAc-β-1,3-Gal-β-1,3-GlcNAc tetrasaccharide glycan under the control of arabinose promoter. **(E)** Production of human-like glycosylated protein using IPTG and arabinose induction. The bar graph represents the total glycoprotein yield, and the line graph illustrates the glycosylation efficiency at different time points, with the positive control protein yield being 58.5 mg/L.

In our previous work, we successfully constructed plasmids pC15-plsg and pIG6-Sia to synthesize the oligosaccharide Gal-β-1,4-GlcNAc-β-1,3-Gal-β-1,3-GlcNAc- and to modify sialylation, respectively, in *E. coli*. This study involved integrating the following three genes or gene clusters related to tetrasaccharide biosynthesis into the *E. coli* genome: 1) the *lsgCDEF* gene cluster derived from *Haemophilus influenzae*, which sequentially extends the tetrasaccharide on the inner surface of the cytoplasmic membrane; 2) the *pglK* gene, which flips the oligosaccharide from the cytoplasm to the periplasmic side of the membrane; 3) the OSTase *pglB* of *C. jejuni*, which transfers the oligosaccharide onto receptor proteins containing the glycosylation recognition sequence DQNAT (Figure 1B).

Following metabolic flux analysis, we prioritized replacing the ECA locus and the *nanKETA* locus (Figure 1C, Supplementary Figure S1A). The former functions as a virulence factor, and the energy and substrates required for its production can be better utilized to promote cell growth or the production of metabolites (Valderrama-Rincon et al., 2012). The latter is involved in the degradation of precursor Neu5Ac in XL1-Blue (Kalivoda et al., 2003) and its deletion (to create the engineered strain denoted XΔK) is considered essential.

Initially, the pC15-plsgΔw plasmid contained the dose-dependent arabinose promoter to enhance glycoprotein expression in the dual-plasmid system. As previous studies have indicated that the ECA native promoter is associated with low expression levels (Englaender et al., 2017; Goormans et al., 2020), this was substituted in the XL1-Blue genome with stronger promoters, thus replacing the *wzzE-yifK* gene cluster in the ECA locus with the *pglB*, *pglK* and *lsgCDEF* genes in combination with the arabinose promoter to establish the XEA strain, as well as replacing the *nanA-nanK* gene cluster in the *nanKETA* locus to establish the XKA strain (Supplementary Figure S1A). The successful construction of these strains was verified through colony PCR and DNA sequencing (Supplementary Table S1, Supplementary Figure S1B).

To assess the stability of strains after genome integration, growth curves were plotted. Notably, strain XKA, which was modified at the *nanKETA* site, exhibited slightly higher growth rates compared to strain XEA, modified at the ECA site, or the wild-type XL1-Blue strain. Nevertheless, the similarity between the growth curves (Supplementary Figure S2A) indicated that neither the knockout of native gene clusters nor the knock-in of the tetrasaccharide synthesis pathway significantly influenced the metabolic load of these strains. Following uptake of plasmid pIG6-FM-Gly encoding glycan receptor proteins, the growth of strains with either single or dual plasmids was investigated (Figure 1D). The XEA-G strain, harboring pIG6-FM-Gly, consistently showed a lower OD_600_ value than the XKA-G strain, which may be attributable to an impact of deletion of the ECA cluster on material exchange and membrane permeability. Meanwhile, strain XL-G harboring plasmids pIG6-FM-Gly and pC15-plsgΔw exhibited the slowest growth rate, suggesting that the metabolic burden of these plasmids may reduce cell growth rate and productivity. With the addition of arabinose every 12 h, the *N*-linked glycosylation efficiency of both XEA-G and XKA-G strains progressively increased to nearly 100% after 42 h of induction (Supplementary Figure S2B, right side of Figure 1E). In contrast, strain XL-G exhibited a glycosylation efficiency of approximately 95% (with a yield of 45 ± 2 mg/L) around 18 h post-induction. However, this efficiency gradually decreased, accompanied by a significant reduction in yield at 48 h post-induction (32 ± 5 mg/L). Notably, the *N*-glycoprotein yield of both XEA-G and XKA-G strains with a single plasmid remained high over the 48 h study, and the XEA-G strain reached a yield of 281 ± 7 mg/L approximately 24 h post-induction, representing a 6.2-fold increase over the XL-G strain (left side of Figure 1E). These results demonstrate the successful construction of glyco-engineered *E. coli* for the efficient biosynthesis of human-like tetrasaccharide glycans.

### Constructing diverse promoter-regulated tetrasaccharide *E. coli* chassis to enhance human-like glycoprotein production

**FIGURE 2:**
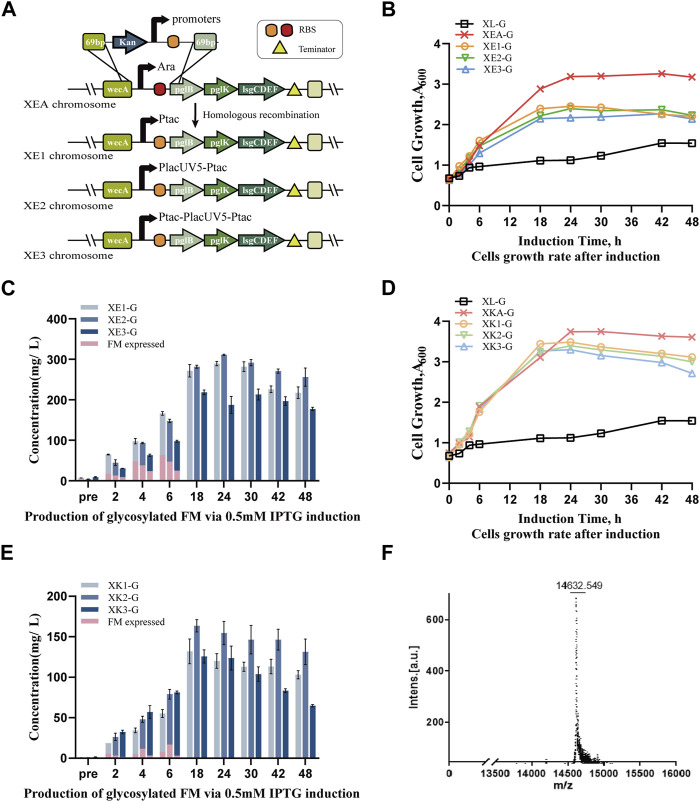
Enhancement of biosynthesizing human-like glycoproteins by promoter optimization in chassis strains. **(A)** Integration of different promoters into the locus between *wecA* and *pglB*. Strain XEA contained the Ara promoter with a matching RBS module, while strains XE1, XE2 and XE3 contained the tac, tac-lacUV5, and tac-lacUV5-tac promoters with matching RBS modules, respectively. **(B)** Cell growth curves of engineered strains XE1-G, XE2-G and XE3-G expressing glycosylated receptor proteins FM-Gly modified by the tetrasaccharide glycan under the control of different promoters (Ptac, PlacUV5-Ptac, Ptac-PlacUV5-Ptac) at the ECA locus, compared with engineered strains XEA-G, XL-G under the control of arabinose promoter. **(C)** Production of human-like glycoproteins in XE1-G, XE2-G and XE3-G strains from 0.5 mM IPTG-induced, plasmid-borne genes, where pink represents the unmodified FM protein. **(D)** Growth curves of engineered strains expressing glycosylated tetrasaccharide-modified FM-Gly, integrated at the *nanKETA* locus: XK1-G, XK2-G and XK3-G under the control of promoters Ptac, PlacUV5-Ptac and Ptac-PlacUV5-Ptac, respectively; and XKA-G and XL-G, under the control of the arabinose promoter. **(E)** Production of human-like recombinant glycoproteins in XK1-G, XK2-G and XK3-G strains from 0.5 mM IPTG-induced, plasmid-borne genes, where pink represents the unmodified recombinant protein. **(F)** MALDI-TOF detection of glycoprotein biosynthesis in the XE2-G strain. Data are presented as mean values +/− SD (n = 3 independent experiments).

To construct cost-effective and stable engineered strains, various synthetic promoters were employed. The tac promoter, a robust hybrid created by merging the trp and lacUV5 promoters, exhibited superior transcriptional activity, surpassing PlacUV5 by 3.3-fold (Deng et al., 2018). Accordingly, they were recombined to generate three distinct promoters ranked from weak to strong: Ptac, PlacUV5-Ptac, and Ptac-PlacUV5-Ptac (Supplementary Figure S3A), which were compared in *N*-glycoprotein expression in this study. Utilizing the Red recombination system (Supplementary Figure S3B), the arabinose promoter in the XEA strain was replaced with different promoter fragments, resulting in the XE1, XE2, and XE3 strains (Figure 2A), which were then transformed with pIG6-FM-Gly, yielding strains XE1-G, XE2-G and XE3-G, respectively.

No significant difference was noted between growth of strains containing plasmids encoding the FM receptor protein induced using the synthetic or arabinose promoters. Nevertheless, the absence of arabinose in the regular medium and the cost associated with periodically adding arabinose were noted.

Given that FM protein expression is regulated by the Lac promoter, varying the concentration of IPTG may influence the balance between protein production and glycosylation modification. Consequently, we induced protein expression using 0.1 mM, 0.5 mM, and 1 mM IPTG and monitored glycosylation efficiency (Supplementary Figure S4). The three strains exhibited a maximum of *N*-glycoprotein yield at 0.5 mM IPTG, with glycosylation efficiency reaching nearly 100% at 10 h post-induction. Notably, with an IPTG concentration of 1 mM, the XE3-G strain exhibited the slowest growth rate and the lowest yield, indicating a higher metabolic burden than in the other strains. When 0.5 mM IPTG was used to further investigate *N*-glycoprotein production under the control of various promoters, strains XE1-G, XE2-G and XE3-G exhibited significantly higher growth rates than strain XL-G, but reduced growth compared to XEA-G. However, the yields of *N*-glycoproteins were considerably higher, reaching approximately 292 ± 3 mg/L, 315 ± 2 mg/L, and 222 ± 3 mg/L with 100% glycosylation efficiency at 18 h post-induction, respectively, roughly 7 times higher than that of XL-G (Figure 2B, C). The lower yield determined for strain XE3-G suggested that incorporating excessive promoters might lead to a higher metabolic load and lower metabolic flux in *E. coli*. Unexpectedly, preliminary glycosylation processes were observed in strains XE1-G, XE2-G and XE3-G even without IPTG induction, indicating that *E. coli* may have an intrinsic regulatory mechanism for utilizing different carbon sources in the medium to synthesize and transfer glycans to the receptor proteins, which will be explored further (Figure 2C, Supplementary Figure S5A).

Additionally, the arabinose promoter in strain XKA was replaced with the three different promoters to create strains XK1, XK2 and XK3. Upon transformation with the plasmid carrying the receptor protein, strains XK1-G, XK2-G and XK3-G exhibited noticeable growth advantages compared to XE2-G (Figure 2B, D). However, their protein yield was lower than that of the ECA replaced strains, particularly in the XE2-G strain (Figure 2C, E), reaching only 135 ± 4 mg/L, 167 ± 5 mg/L, and 131 ± 1 mg/L, respectively, although they initially exhibited almost 100% glycosylation efficiency (Figure 2D, E, Supplementary Figure S5B). Subsequent MALDI-TOF detection confirmed the above findings, identifying the peak at 14632.549 as *N*-glycosylated FM proteins modified with the Gal-β-1,4-GlcNAc-β-1,3-Gal-β-1,3-GlcNAc- tetrasaccharide (Figure 2F). In conclusion, we found that integrating the PlacUV5-Ptac promoter at the ECA site, namely, strain XE2, was the best choice as the novel *E. coli* chassis.

### Applying “plug-and-play” tetrasaccharide chassis cells to accomplish efficient sialylation on *N*-glycoproteins using a single plasmid harboring the target protein and sialylation modifying pathway

**FIGURE 3:**
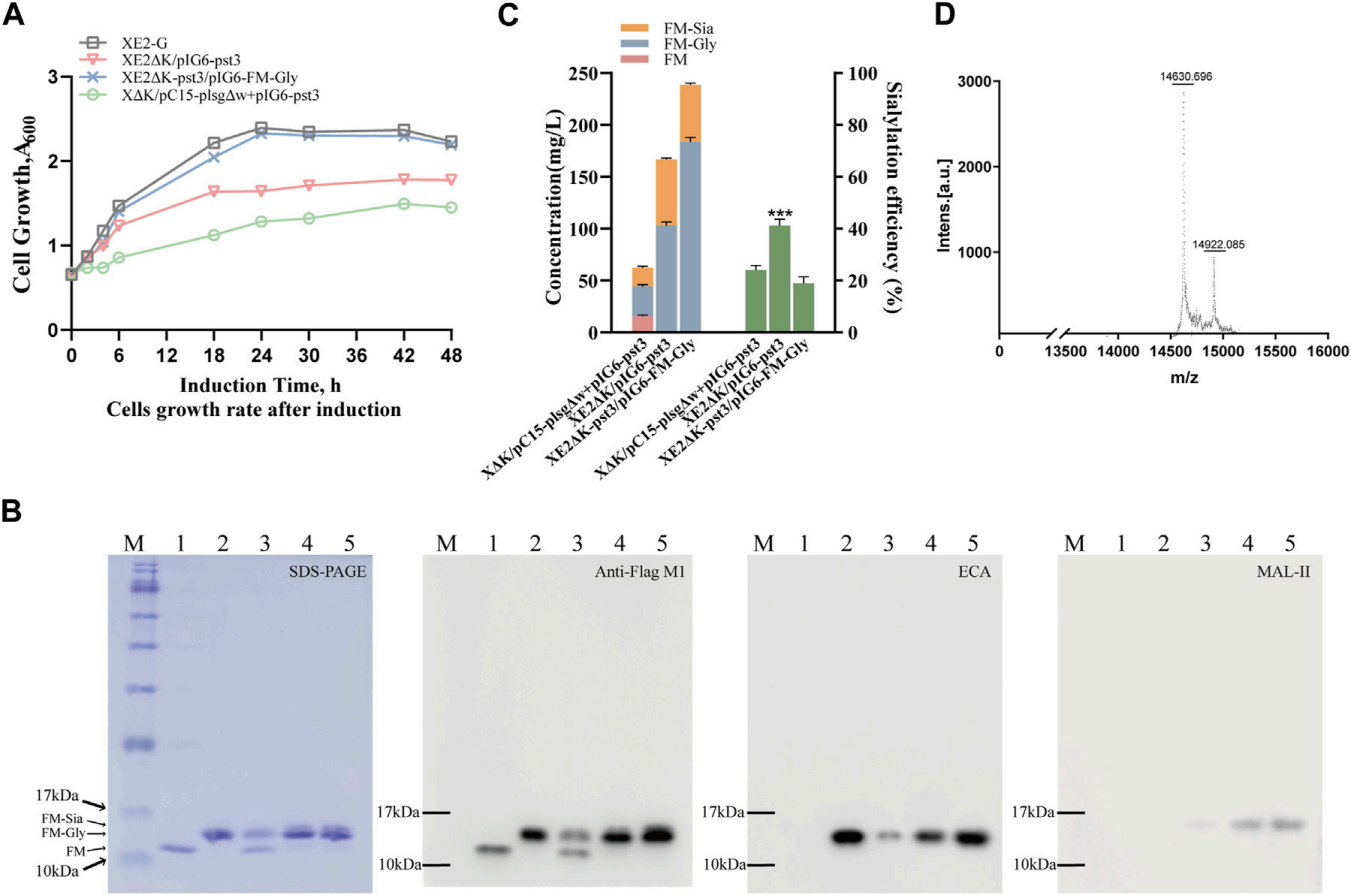
Production of human-like sialylated *N-*glycoproteins in glyco-engineered *E. coli*. **(A)** Growth curves of strains expressing α-2,3-sialyltransferase pst3 in different expression modes before and after genomic integration. **(B)** SDS-PAGE with Coomassie brilliant blue staining, followed by immunoblotting using anti-Flag M1 antibody and lectin blotting using Gal-β-1,4-GlcNAc-specific *Erythrina cristagalli* lectin (ECA) and Neu5Ac-α-2,3-Gal/GalNAc-specific *Maackia amurensis* lectin II (MAL-II). Lanes 1-2 represent purified unmodified protein FM and glycosylated modified protein FM-Gly, while Lanes 3-5 show sialylated protein FM-Sia expressed from dual-plasmid XΔK/pC15-plsgΔw + pIG6-pst3, single plasmid XE2ΔK/pIG6-pst3 and genomic XE2ΔK-pst3/pIG6-FM-Gly, respectively. **(C)** Quantitative analysis of glycosylation patterns of the strains depicted in Fig. B and three independent growth curves were used to generate the stacked histograms representing the average glycosylation efficiency with the following colors: pink (FM), blue (FM-Gly), orange (FM-Sia). Green represents the average sialylation efficiency, and the modification efficiency of sialylated proteins depends on the system used. **(D)** MALDI-TOF detection of glycoproteins biosynthesis in strain XE2ΔK/pIG6-pst3. Data are presented as mean values +/− SD (n = 3 independent experiments). ***p < 0.001, student’s t-test.

To synthesize sialylated *N*-glycoproteins with an α-2,3-linkage, the α-2,3-sialyltransferase 2,3SiaTpph (A151D) variant from *Photobacterium phosphoreum* JT-ISH-467 (Mertsch et al., 2020) was cloned to produce plasmid pIG6-pst3, which carries the dual-expression cassette of the receptor protein and the CMP-sialic acid synthesis pathway, under the control of the lac promoter and the P constitutive promoter, respectively. To further investigate efficient sialylation pathways, the *nanKETA* gene cluster in strain XE2 was either knocked out or replaced with the genes of α-2,3-sialyltransferase and neuBCA synthetases, resulting in strains XE2ΔK and XE2ΔK-pst3 (Supplementary Figure S6A). Subsequently, the pIG6-FM-Gly plasmid was introduced into strains XE2ΔK-pst3, using the two-plasmid-harboring strain XΔK/pC15-plsgΔw + pIG6-pst3 and the single-plasmid-containing strain XE2ΔK/pIG6-pst3 for comparative analysis at 0.5 mM IPTG concentration. Notably, all three strains exhibited a slower growth rate and reduced yield compared to XE2-G (Figure 3A, Supplementary Figure S6B, C), indicating an elevated metabolic burden associated with sialylation.

Compared with the two-plasmid-harboring strain XΔK/pC15-plsgΔw + pIG6-pst3, the single-plasmid-containing strains XE2ΔK/pIG6-pst3 and XE2ΔK-pst3/pIG6-FM-Gly achieved 100% glycosylation efficiency directly after induction (Supplementary Figure S6B, C), and the yield of sialylated protein increased to 65 mg/L and 57 mg/L, respectively. It is noteworthy that the sialylation efficiency of the former strain increased by 53%, whereas the latter exhibited no improvement (Figure 3B, C). Furthermore, upon detection *via* MALDI-TOF, the mass at 14922.215 corresponded to further proteins modified by the sialylated oligosaccharide Neu5Ac-α-2,3-Gal-β-1,4-GlcNAc-β-1,3-Gal-β-1,3-GlcNAc- (Figure 3D), indicating that approximately 30%–40% of α-2,3-sialylated glycosylation modifications exist in the glycosylated protein. These findings suggest the successful application of tetrasaccharide chassis cells to obtain homogeneously sialylated *N*-glycoproteins using a single plasmid carrying both the target protein and sialylation pathway, which will be convenient in producing other sialylated glycoproteins with different linkages.

## Discussion

In this study, a plug-and-play *E. coli* chassis was successfully constructed to produce homogeneously *N*-glycosylated proteins with human-like sialylation in the periplasm. This was accomplished using a single plasmid to direct α-2,3-linked sialylation, without the need for costly arabinose or unstable Neu5Ac in the medium. Importantly, this method offers a convenient and cost-effective approach to obtaining *N*-glycoproteins with α-2,3-sialylation linkages and represents a significant advance over both the eukaryotic *N*-glycosylation modification system and chemical enzymatic methods that have been reported to date, both *in vivo* and *in vitro* (Poljak et al., 2018; Prabhu et al., 2021). As complex glycoforms can interfere with the folding of *N*-glycoproteins, an engineering strategy, GlycoDelete, which shortens the *N*-glycosylation pathway, has been reported which produced glycoproteins in 293T cells that were terminally α-2,3-sialylated with Neu5Ac-α-2,3-Gal-β-1,4-GlcNAc- (consistent with our glycan terminus) (Meuris et al., 2014; Kozak et al., 2020). The modification resulted in a reduced initial serum clearance rate and no new immunogenicity. Other researchers have employed multiple plasmids for the economic production of similar sialylated glycoproteins in *E. coli* but faced challenges due to the instabilities of multi-plasmid *E. coli* systems (Keys et al., 2017; Zhu et al., 2020).

It is worth mentioning that our constructed glycoengineering strains effectively alleviated the instability of plasmid systems, although the mechanism by which this is achieved requires further exploration. Nevertheless, this improvement led to a substantial increase in the yield of *N*-glycosylated recombinant proteins compared to dual-plasmid systems (Figure 1E). Initially, a relatively strong and dose-dependent arabinose promoter was used to regulate tetrasaccharide synthesis in the dual-plasmid system, leading to the glycosylation efficiency reaching its peak quickly after induction but the OD value remaining low. While extending the induction time achieved a slight increase in OD, this was countered by a significant decrease in glycosylation efficiency, indicating an imbalance between receptor protein expression and glycan chain synthesis and transfer to the receptor. Subsequently, integration into the host genome of the tetrasaccharide synthesis cluster *lsgCDEF* under the control of differing strength promoters was used to create a stable strain suitable for integration of additional glycan synthesis modules, such as the sialylation pathway. Despite a delay in the glycosylation efficiency reaching 100% after induction, which might be attributable to the reduced copy number of the glycosyltransferase, extending the induction period resulted in high-density growth in all strains, accompanied by 4–6 times increase in glycoprotein yield. To increase the rate of glycosylation efficiency and increase yields, our ongoing strategy entails refining the functionality of each module by investigating more diverse ribosome-binding sites and promoters, as well as prioritizing strain screening, based on the success of achieving gram-level protein yields in the *E. coli* B series strains.

Our strategy for constructing the plug-and-play *E. coli* chassis relies on balancing two distinct pathways: the genome-integrated tetrasaccharide synthesis pathway and the plasmid-encoded sialylation pathway. While plasmids have been widely used to construct metabolic pathways, their application for industrial glycoprotein production has been hindered by their inherent instability and the associated metabolic burden, especially when expression is controlled by strong promoters (Glasscock et al., 2018). Therefore, we leveraged pathways of plasmids to achieve homogeneous and cost-effective production in a stable chassis strain. This initially involved genome integration of the *pglB*, *pglK* and *lsgCDEF* gene clusters, aiming to mitigate instability issues associated with plasmids as well as adjusting enzyme expression levels for effective oligosaccharide synthesis. Specifically, integrating the tetrasaccharides Gal-β-1,4-GlcNAc-β-1,3-Gal-β-1,3-GlcNAc- synthesis pathway into the genome combined with systematical optimization of promoters has also ensured the normal functioning of microbial glycosyltransferases and other related enzymes to meet the needs of glycan synthesis from a single copy of the gene. Subsequently, the pIG6 vector for sialylation was conveniently transferred to the tetrasaccharide synthesis chassis, thereby creating a plug-and-play *E. coli* to accomplish α-2,3-sialylation. This approach makes possible the efficient modification of either different sialylation linkages of α-2,3/α-2,6/α-2,8 in human glycoepitopes or various receptor proteins simply by plasmid transfer. However, with our subsequent integration of *pst3* and *neuBCA* gene clusters into the genome, a larger yield of sialylated glycoproteins could be obtained albeit with lower sialylation efficiency, which warrants future exploration (Figure 3).

Importantly, we observed that inducing the full sialylation pathway in the glyco-engineered strain negatively impacted host cell growth, leading to a significantly reduced yield of the sialylated protein. Our previous study of the plasmid system also revealed a substantial decrease in the yield of sialylated protein (Zhu et al., 2020), with similar trends observed in the production of *N*-glycosylated proteins with the human-like tetrasaccharide glycan and terminal α-2,3-linked sialylation, reaching yields of 320 mg/L and 65 mg/L, respectively. To alleviate this metabolic burden, we propose to further optimize gene expression levels through engineering promoters and ribosome-binding sites to better balance the protein expression, LLO production, and glycosylation processes. As improving the terminal sialylation efficiency remains a challenge and other studies have suggested that overexpression of *neuA*, to supplement CMP-Neu5Ac, and alkaline phosphatase can decrease the efficiency of the sialidase enzyme, thereby increasing its sialyltransferase efficiency (Kang et al., 2015; Zhang et al., 2022; Zhao et al., 2023), we will focus on incorporating a stronger promoter into the *neuBCA* gene cluster, as well as screening other α-2,3-sialyltransferases to optimize sialylation.

In conclusion, this work provides a convenient and cost-effective *E. coli* chassis for the production of a human-like *N*-glycosylated recombinant antibody with terminal sialylation. Ongoing optimization will further improve homogeneous sialylation, potentially expanding into different linkages (α-2,3/α-2,6/α-2,8) in sialylated glycoepitopes associated with human disease. The technological platform also establishes the groundwork to investigate the impact of homogeneous glycans on the structure and function of recombinant antibodies, and to advance the industrial production of a diverse range of sialylated glycoprotein drugs.

## Data Availability

The raw data supporting the conclusion of this article will be made available by the authors, without undue reservation.
