# Supplementary material for: Construction of an Escherichia coli chassis for efficient biosynthesis of human-like N-linked glycoproteins
Source: Front Bioeng Biotechnol. 2024 Mar 20;12:1370685. doi: 10.3389/fbioe.2024.1370685 (PMC10987854; doi:10.3389/fbioe.2024.1370685)

Supplementary Material

Construction of an *Escherichia coli* Chassis for Efficient Biosynthesis of Human-like *N*-linked Glycoproteins

Zixin Bao^1†^, Yuting Gao^1†^, Yitong Song^1^, Ning Ding^1,2^*, Wei Li^1^, Qiong Wu^1^, Xiaomei Zhang^1^, Yang Zheng^1^, Junming Li^3^*, Xuejun Hu^1,2^*

^1^ Academic Centre for Medical Research, Medical College, Dalian University, Dalian, China

^2^ Dalian Key Laboratory of Oligosaccharide Recombination and Recombinant Protein Modification, Dalian, China

^3^ Department of Clinical Laboratory, Yantai Yuhuangding Hospital, Yantai, China

*** Correspondence:**

Ning Ding

[dingning@dlu.edu.cn](mailto:dingning@dlu.edu.cn)

Junming Li

[junming1616@126.com](mailto:junming1616@126.com)

Xuejun Hu

[huxuejun@dlu.edu.cn](mailto:huxuejun@dlu.edu.cn)

^†^These authors have contributed equally to this work.

# Supplementary Table 1. Plasmids and primers in this study.

| Plasmids | characteristics | Reference or source |
| --- | --- | --- |
| pC15-plsg | “*N*-glycosylation pathway,” includes rbs – *pglB* – rbs1 – *wecA* – *wzzE* rbs2 –*pglK* – *ompA* rbs – *lsgCDEF* – *rrnB* terminator, all under the control of an arabinose promoter, Cm^R^; vector backbone is pC15. rbs1 and rbs2 are from artificial synthesis sequences based on the rbs (core sequence: AGGA) of pET28a (Novagen) | (Zhu et al., 2020) |
| pC15-plsgΔw | “*N*-glycosylation pathway,” CM, KAN, KAN-rbs – *pglB* – rbs1 – *wzzE* rbs2 –*pglK* – *ompA* rbs – *lsgCDEF* – *rrnB* terminator, all under the control of an Arabinose promoter, Cm^R^; vector backbone is pC15. rbs1 and rbs2 are from artificial synthesis sequences based on the rbs (core sequence: AGGA) of pET28a (Novagen) | This study |
| pIG6-FN3-Gly-1 | glyco-tagged FN3 (MK355444) expression vector, includes lac promoter –*ompA* leader peptide – *FN3* – DQNAT sequon – 6×His – terminator, Amp^R^; vector backbone is pIG6 | (Ding et al., 2017) |
| pIG6-FM-Gly | glyco-tagged Fn3 3.4.4 (Sirois et al., 2020) expression vector, includes lac promoter - *OmpA* leader peptide - DYKD flag - *Fn3 3.4.4* - DQNAT sequon - 6×His-terminator, Amp^R^; vector backbone is pIG6 | This study |
| pIG6-pst3 | “sialylation pathway,” includes Lac promoter – *ompA* leader peptide – *Fn3 3.4.4* – DQNAT sequon – T7 terminator –P regulatory region – 2,3SiaTpph (A151D) – *ompA* rbs – *neuBCA*, Amp^R^; vector backbone is pIG6. P regulatory region: the upstream sequence of the *N*-glycosylation *pgl* locus from *C. jejuni* | This study |

| Primers | Sequences (5′-3′) |
| --- | --- |
| pglBF1 | GAGGAATTACATATGATGTTGAAAAAAGAG |
| pglBR1 | AAAGTTTTTTTAGCATCACATCCTCATTTAAATTTTAAGTTTAAAAACCTTAGCATC |
| pglKF2 | GATGCTAAGGTTTTTAAACTTAAAATTTAAATGAGGATGTGATGCTAAAAAAACTTT |
| pglKR2 | GCTTTAGAAAAAGCTTCACTTTGTGC |
| ECA-lsg-F | TGGATATTCGTGGGTTGTTTCGTACCTTGTGGGCTGGGAAGCTATGGATTATTGGCATGGGGCTGGCGTGTAGCGCAGGCCGACTACAAAGAT |
| ECA-lsg-R | CACGCTGGCACTGTAGACGTAGACAAACACACGCTGCGGATTGGGAATGATGTAGTTCAGGCATGAGCCTTCATCGTATTTCCTATTTACGT |
| nanKETA-lsg-F | TGGATATTCGTGGGTTGTTTCGTACCTTGTGGGCTGGGAAGCTATGGATTATTGGCATGGGGCTGGCGTGTAGCGCAGGCCGACTACAAAGAT |
| nanKETA-lsg-R | AACCTGCATCATGGCGGTAATGCGCCGCCAGTAAATCAACATGAAATGCCGCTGGCTCCTGCGCCAGATTTCATCGTATTTCCTATTTACGT |
| nanKETA-pst3-F | AATTATTGATTCGGCGGATGGTTTGCCGATGGTGGTGTACAACATTCCAGCCCTGAGTGGGGTAAAACTTGTCAAACATGAGAATTAATTCCG |
| nanKETA-pst3-R | AACCTGCATCATGGCGGTAATGCGCCGCCAGTAAATCAACATGAAATGCCGCTGGCTCCTGCGCCAGATTTATTTTTTCCATATCTGTTCAACC |
| Homo-1-3P-R | CTTTAAGAAGGAGATATACCATGTTGAAAAAAGAGTATTTAAAAAACCCTTATTTAGTTTTGTTTGCGATGATTATATTAGCTTATGTTTTTAGTGTATTTTGCAGGTTTTATTGGGTTTGGTGGGCAAG |
| JD-ECA-F1 | GCAGACTGCGTAGAAATCGTGG |
| JD-ECA-R1 | CAGGATTGAGCGGAACGGATG |
| JD-nanKETA-F1 | CTTGCGGCATCGGCTAAACG |
| JD-nanKETA-R1 | CCAGCGTTAACGCGTCCTG |
| JD-pglB-R | CATTAAACTCACTTGCCCACCAAACCCAAT |

**REFERENCES**

Ding, N., Yang, C., Sun, S., Han, L., Ruan, Y., Guo, L., et al. (2017). Increased glycosylation efficiency of recombinant proteins in Escherichia coli by auto-induction. *Biochem Biophys Res Commun* 485(1)**,** 138-143. doi: 10.1016/j.bbrc.2017.02.037.

Sirois, A.R., Deny, D.A., Li, Y., Fall, Y.D., and Moore, S.J. (2020). Engineered Fn3 protein has targeted therapeutic effect on mesothelin-expressing cancer cells and increases tumor cell sensitivity to chemotherapy. *Biotechnol Bioeng* 117(2)**,** 330-341. doi: 10.1002/bit.27204.

Zhu, J., Ruan, Y., Fu, X., Zhang, L., Ge, G., Wall, J.G., et al. (2020). An Engineered Pathway for Production of Terminally Sialylated N-glycoproteins in the Periplasm of Escherichia coli. *Front Bioeng Biotechnol* 8**,** 313. doi: 10.3389/fbioe.2020.00313.

# Supplementary Figure 1. Construction of glyco-engineered *E. coli* for efficient biosynthesis of human-like tetrasaccharide glycans. (A) Genomic replacement of native oligosaccharide ECA and *nanKETA* gene clusters in *E. coli* XL1-Blue genome with the biosynthesis pathways of human-like tetrasaccharides and sialic acid, yielding strains XEA and XKA. (B) Integration of tetrasaccharide glycans into the locus between *wecA* and *yifk*. M: DNA marker; lane 1: *wzzE‑yifk* fragment in strain XL1-Blue; lane 2: *Kan-Ara‑pglB-pglK‑lsgCDEF* fragment in strain XEA-1; lane 3: *Ara‑pglB-pglK‑lsgCDEF* fragment in strain XEA; primers lsg F and lsg R were used for all PCR verification. On the right are the sequencing results of the recombinant strains in lane 3.


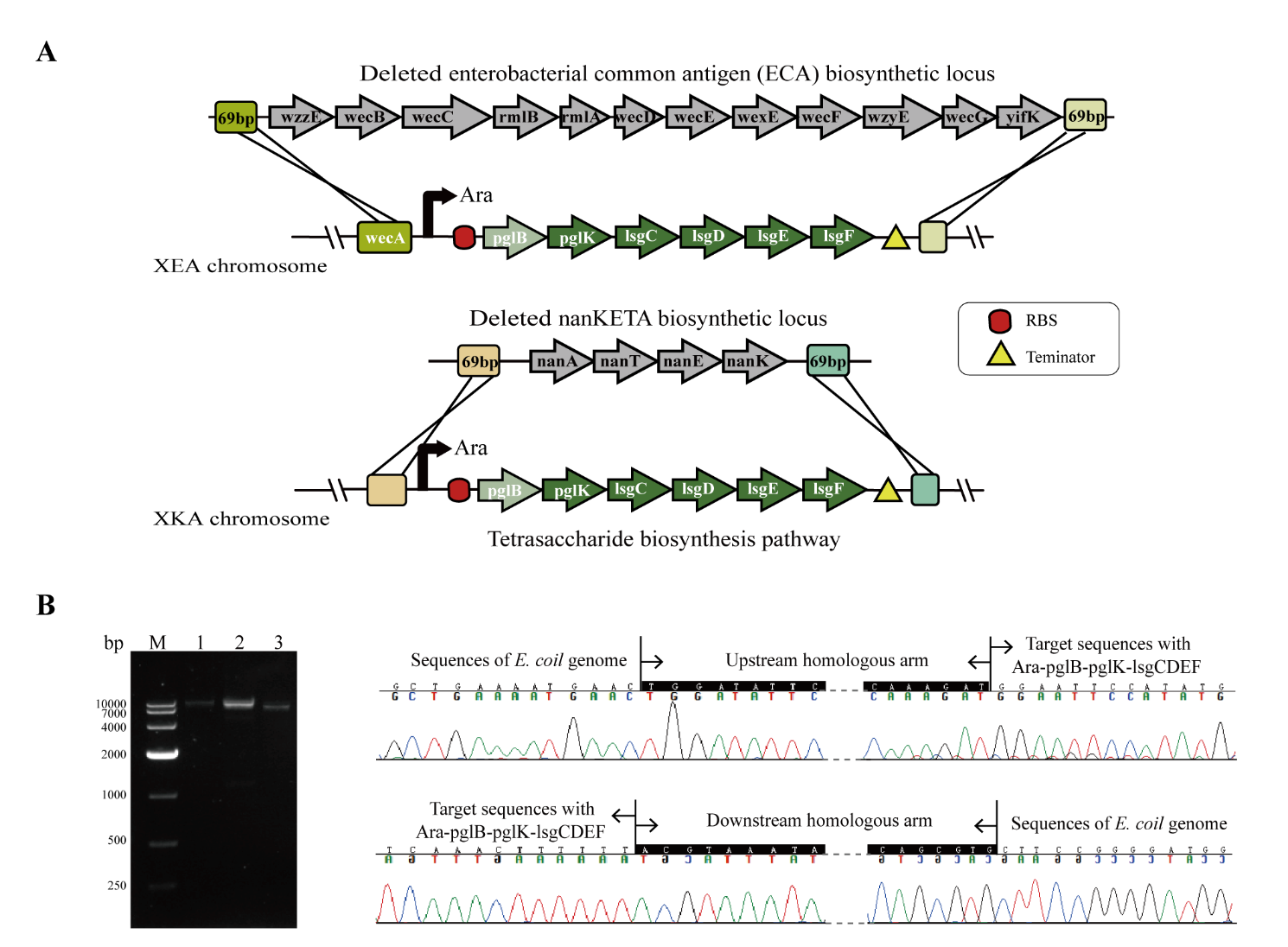


**Supplementary Figure 2.** Site-specific genome integration of tetrasaccharide biosynthesis pathway at ECA and *nanKETA* sites for producing human-like glycoproteins. (A) Growth curves of engineered *E. coli* strains. (B) Western blot analysis of post-modified FM protein expression. At specified induction time points, *E. coli* cell lysates were probed using an anti-FLAG M1 antibody, and the protein yield was measured to determine the optimal expression conditions. FM refers to the purified unmodified protein, and Gly refers to the purified *N*-glycosylated protein.


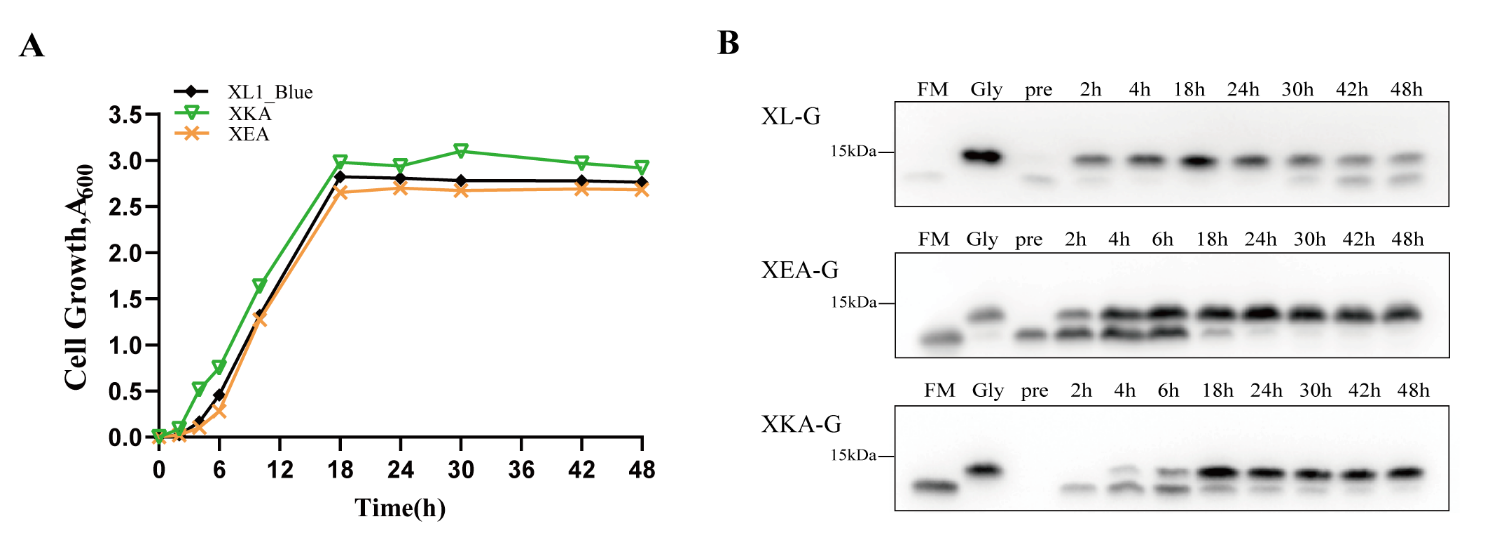


**Supplementary Figure 3.** Promoter sequences and schematic depicting promoter-directed replacement by cloning integration technology. (A) Demonstrating three promoter sequences, the two core promoter sequences are separated by an interval sequence (TGTGG), giving the RNA polymerase enough space for binding. Following the sequence of the last tac promoter is another interval sequence (TGTGG), followed by the lac operon sequence (lac operator) which serves as the binding site for the inducible promoter tac repressor. Following the operator sequence, there is also a ribosome recognition site (AAGGAG), and space reserved for ribosome binding. (B) The Kan fragment containing flanking FRT sites was cloned upstream of the target gene synthesis pathway. The promoter fragment containing the homologous arm was amplified by PCR, and the construction of an engineered strain of *E. coli* with an inducible promoter regulating the synthesis pathway of the target gene was completed by homologous recombination.

**
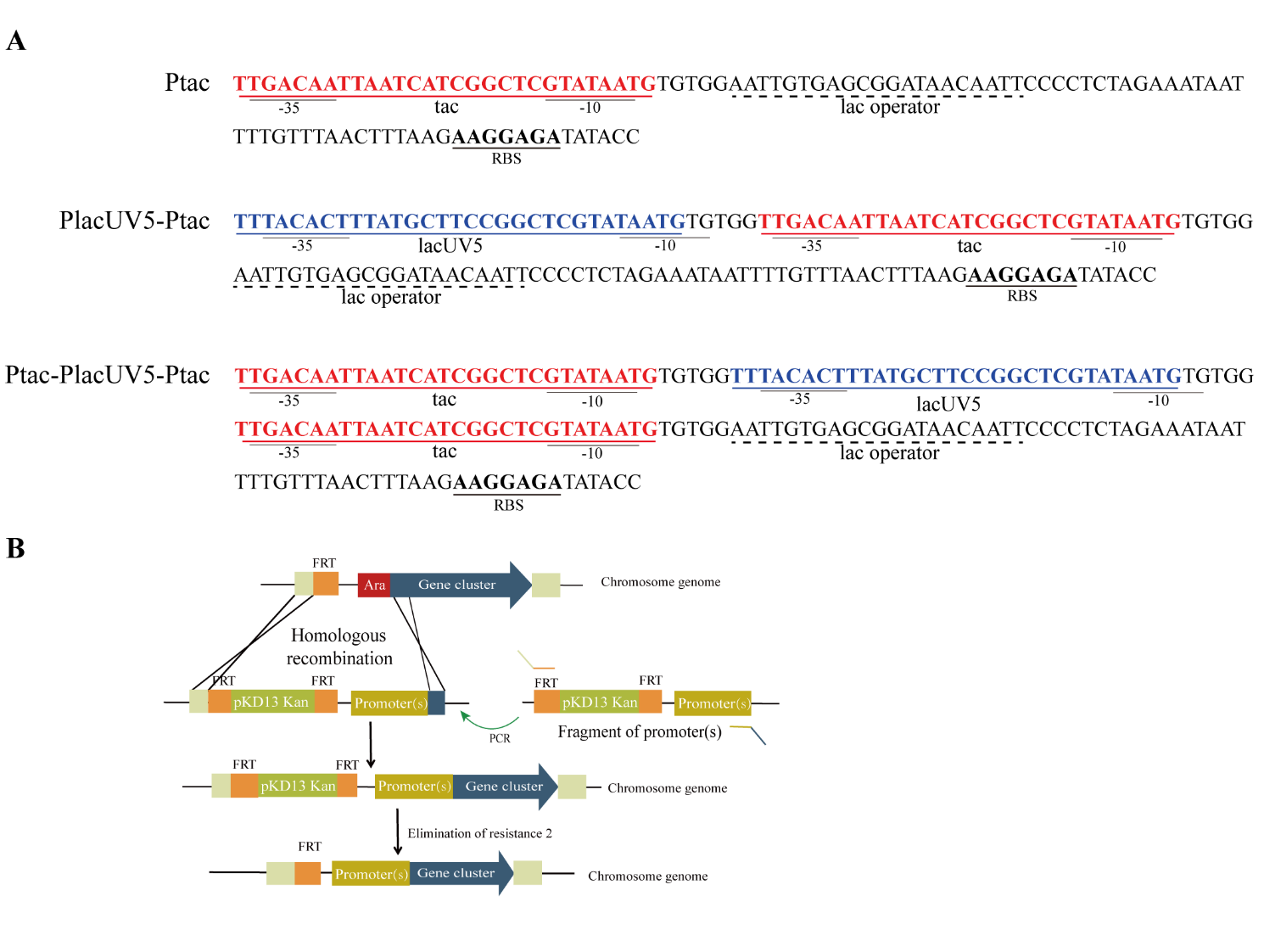
**

**Supplementary Figure 4.** Genome integration of tetrasaccharide biosynthesis pathway at ECA site to produce human-like glycoproteins under the control of Ptac, PlacUV5-Ptac and Ptac-PlacUV5-Ptac promoters. (A) Western blot analysis of the modified FM protein with induction by different IPTG concentrations to determine the glycosylation efficiency at different time points. (B) Growth curve and (C) Western blot analyses corresponding to (A).


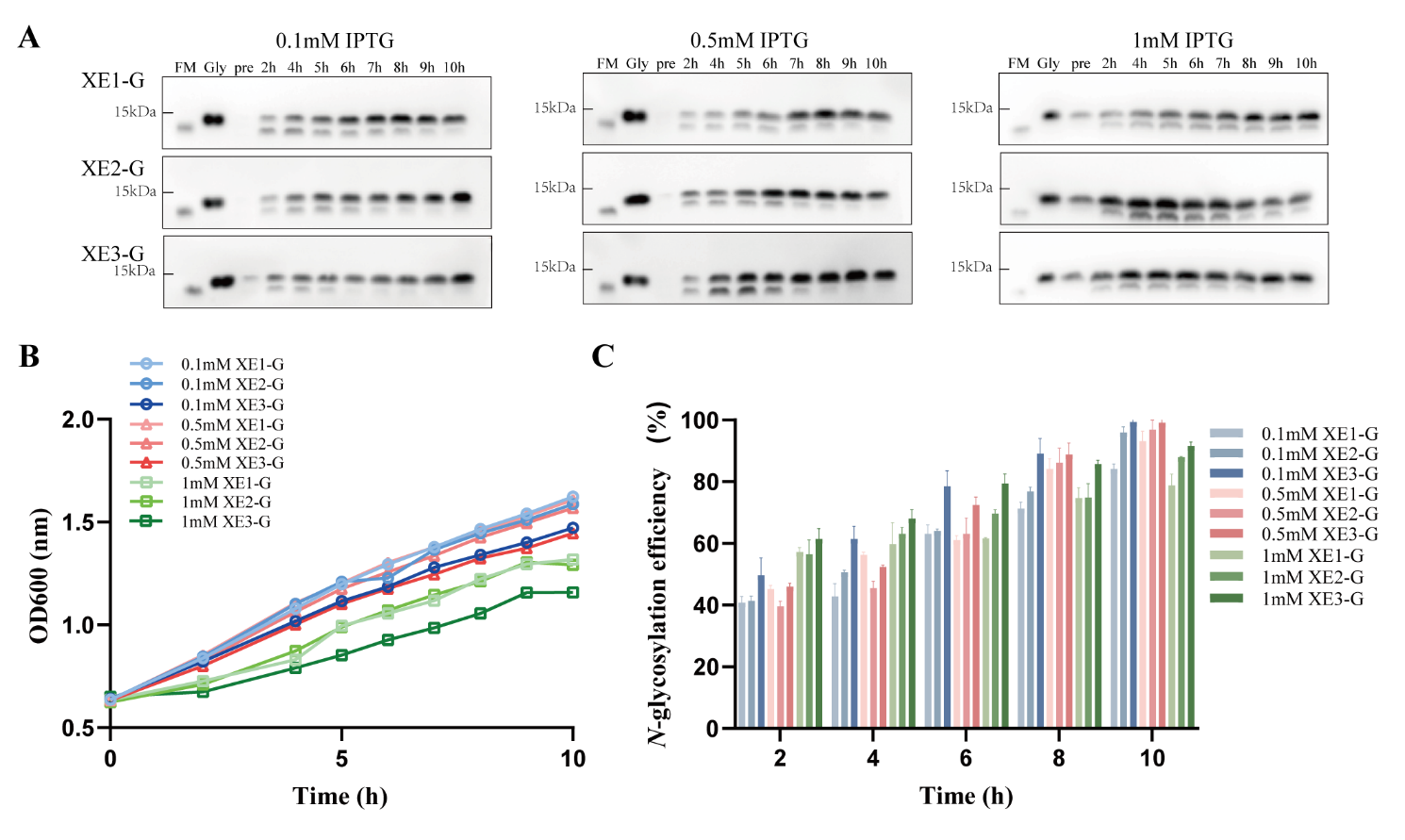


**Supplementary Figure 5.** Comparative analysis of glycoproteins expressed under the control of different strength promoters from genes integrated at ECA or *nanKETA* sites. Western blot detection of *N*-glycoproteins from the (A) ECA and (B) *nanKETA* sites with Ptac, PlacUV5-Ptac and Ptac-PlacUV5-Ptac promoters.


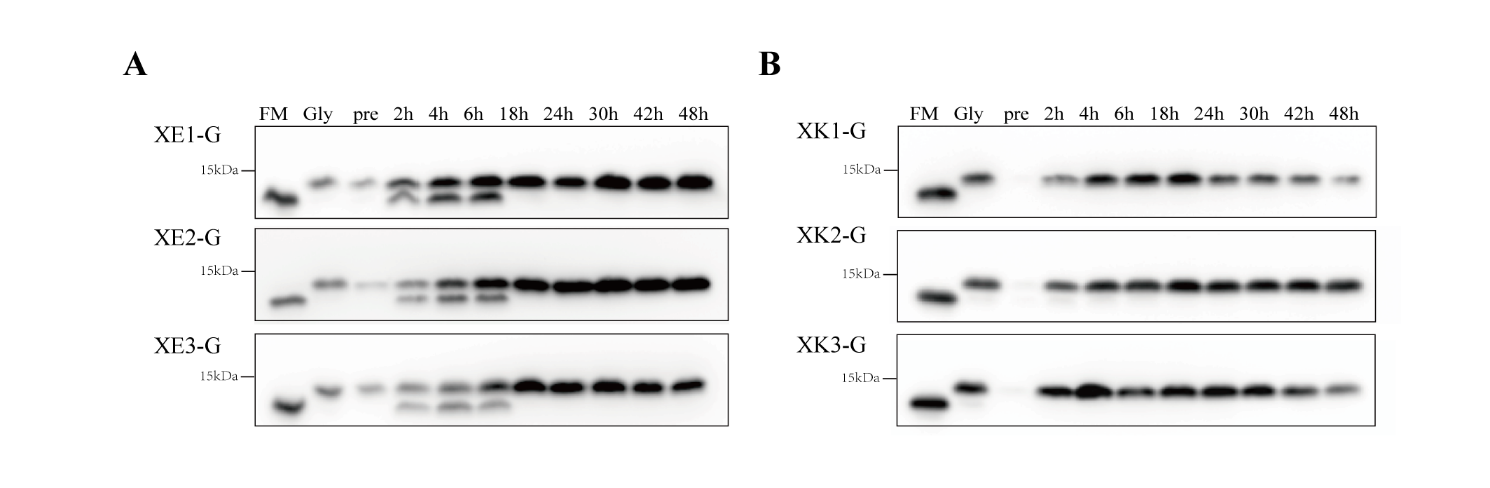


**Supplementary Figure 6.** Sialylation of recombinant proteins facilitated by α-2,3-sialyltransferase. (A) The *nanKETA* gene cluster in strain XE2 was knocked out or replaced with the α-2,3-sialyltransferase *pst3* and sialic acid biosynthetic pathway genes *neuB*, *neuC* and *neuA*, yielding strains XE2ΔK and XE2ΔK-pst3. (B) Western blot detecting the expression of FM-Sia using three different strains: XΔK/pC15-plsgΔw+pIG6-pst3, XE2ΔK/pIG6-pst3 and XE2ΔK-pst3/pIG6-FM-Gly. (C) The corresponding glycoprotein production analysis for Figure B, where pink represents the unmodified recombinant protein, and the positive control protein yield is 156.4 mg/L.


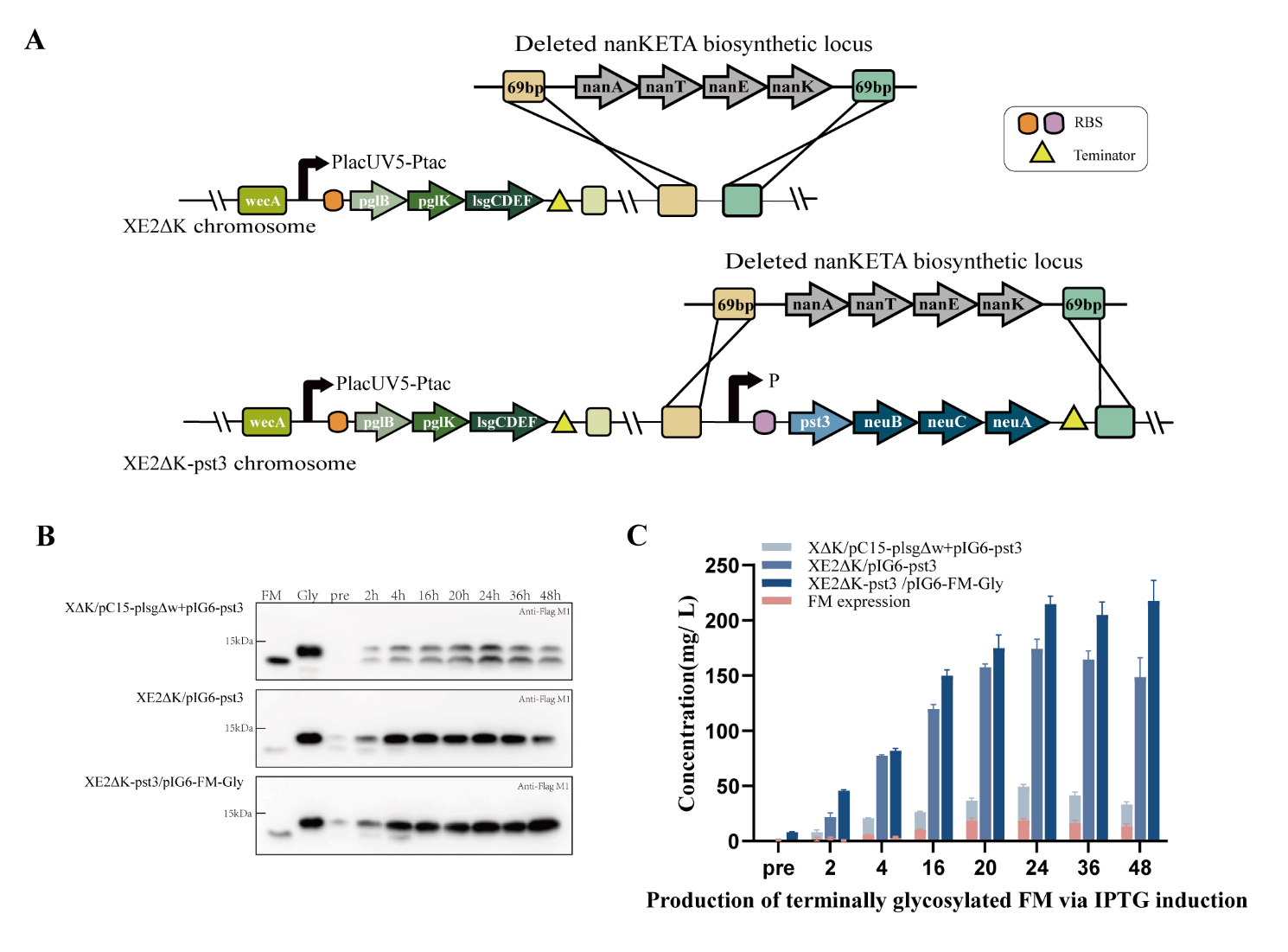

Supplement: Supplementary file 1 [file DataSheet1.docx]
